# Supplementary material for: Usage of Cell-Free Protein Synthesis in Post-Translational Modification of μ-Conopeptide PIIIA
Source: Mar Drugs. 2023 Jul 25;21(8):421. doi: 10.3390/md21080421 (PMC10455749; doi:10.3390/md21080421)
Supplement: Supplementary file 1 [file marinedrugs-21-00421-s001.zip › marinedrugs-2413041-supplementary.pdf]

Figure S1. Gel image of recombinant plasmids pET-SUMO-H, pET-SUMO-C and pET-SUMO-PDI for the expression of hydroxylase (H), glutaminyl cyclase (C) and protein disulfide isomerise (PDI) digested by restriction enzyme. Lanes 1~3: Identification of recombination plasmids pET-SUMO-H, pET-SUMO-C and pET-SUMO-PDI digested by EcoR I and Mlu I, individually, M: 1 kb DNA Ladder.

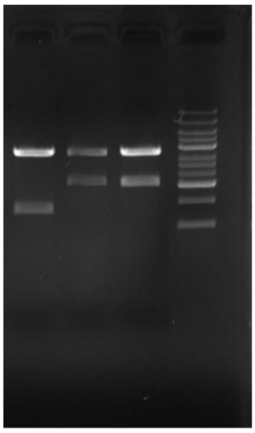

Figure S2. 15% SDS-PAGE analysis of recombinant pET-SUMO-H, pET-SUMO-C and pET-SUMO-PDI expressed in *Escherichia coli* BL21(DE3) pLysS cells and purified by HisPur™ Ni-NTA affinity chromatography column. PM: Multicolor Low Range Protein Ladder (Thermo Scientific 26616). a, recombinant expression of pET-SUMO-H induced by 0.1 mM IPTG at 16°C for 8 h, and then at 28 °C for 10 h; Lane 1: washed fractions of recombinant SUMO-H using 25 mM imidazole; Lane 2~3: Eluted fractions of recombinant SUMO-H using 125 mM and 250 mM imidazole, individually. b, recombinant expression of pET-SUMO-C induced by 0.1 mM IPTG at 16°C for 8 h, and then at 28 °C for 10 h; Lane 1, uninduced recombinant plasmid pET-H as positive control; Lanes 2~3, washed fractions of recombinant SUMO-C using 25 mM imidazole; Lane 4: Eluted fractions of recombinant SUMO-C using 250 mM imidazole. c, recombinant expression of pET-PDI induced by 1 mM IPTG at 37°C for 4 h. Lane 1, uninduced pET-32a (+) as negtive control; Lane 2, uninduced recombinant plasmid pET-H as positive control; Lanes 3-4, washed fractions of recombinant SUMO-PDI using 25 mM imidazole; Lane 5, washed fractions of recombinant SUMO-PDI using 50 mM imidazole; Lane 6, washed fractions of recombinant SUMO-PDI using 125 mM imidazole; Lane 7: Eluted fractions of recombinant SUMO-PDI using 250 mM imidazole.

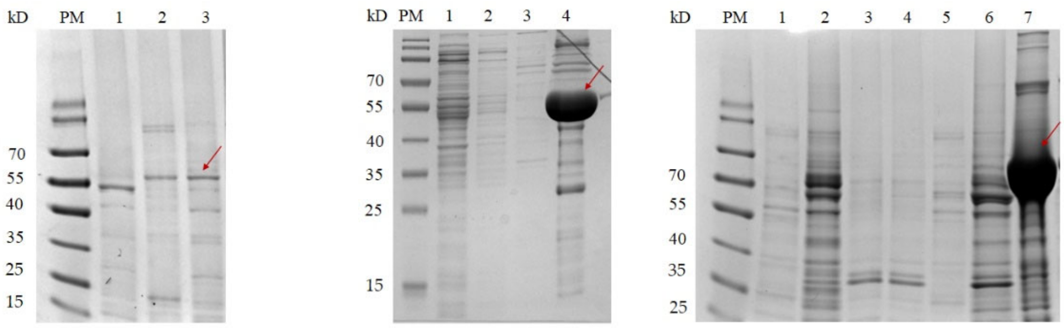

(a) (b) (c)

Figure S3. Construction of pET-p3a3-H expression vector. (a) The gel image of PCR fragments of p3a3 (left) and P4H (right); (b) The gel image of plasmid pET-p3a3 (left) and pET-p3a3-H(right) digested by *Nde* Ⅰ and *Xho* Ⅰ.

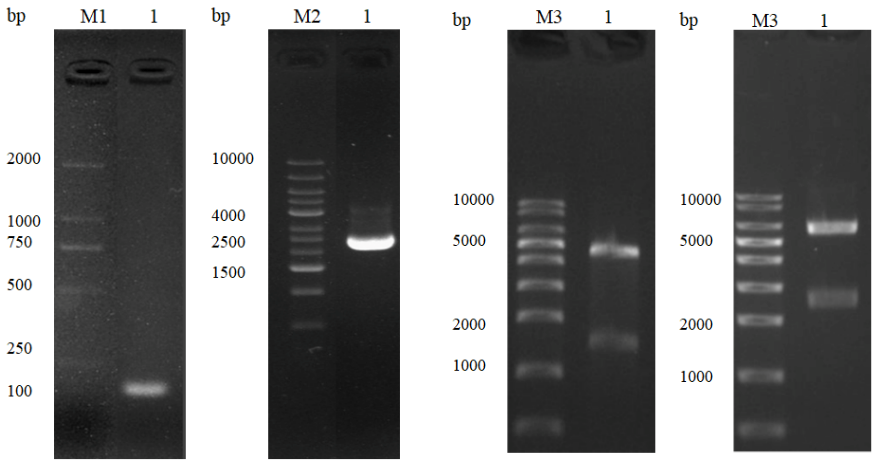

(a) (b)

Table S1. CECF Reaction

Components	Volume(μL)
Cell extracts	25
Reaction Mix	7
Amino Acid Mix	7
Methionine	1
pI-p3a1-2.4d/ pI-p3a2-2.4d/ pI-p3a2-2.4d	0.5
ddH_2O	5
